# Biplanar or Monoplanar Prostate Biopsy: Should Transrectal and Transperineal Approaches be Combined for Prostate Cancer Detection?

**DOI:** 10.1590/S1677-5538.IBJU.2024.0630

**Published:** 2025-01-03

**Authors:** Zeng Zhou, Tiewen Li, Yichen Zhang, Xuehao Zhou, Xiaohai Wang, Di Cui, Yiping Zhu, Chenyi Jiang, Wenhuan Guo, Bangmin Han, Yuan Ruan

**Affiliations:** 1 Shanghai Jiao Tong University School of Medicine Shanghai General Hospital Department of Urology Shanghai China Department of Urology, Shanghai General Hospital, Shanghai Jiao Tong University School of Medicine, Shanghai, China; 2 Shanghai Jiao Tong University School of Medicine Shanghai Ninth People's Hospital Department of Pathology Shanghai China Department of Pathology, Shanghai Ninth People's Hospital, Shanghai Jiao Tong University School of Medicine, Shanghai, China

**Keywords:** Prostatic Neoplasms, Ultrasound, High-Intensity Focused, Transrectal, Diagnosis

## Abstract

**Purpose:**

The accurate diagnosis of prostate cancer (PCa) remains challenging, particularly because standard biopsy techniques do not routinely include anterior zone, leading to potential missed diagnoses in this region. This study evaluates the accuracy and safety of biplanar stereotactic biopsy for diagnosing anterior clinically significant PCa (csPCa).

**Materials and Methods:**

After propensity score matching analysis, data from 256 patients were retrospectively analyzed, including 128 in the biplanar group (transrectal targeted biopsy with transperineal systematic biopsy) and 128 in the monoplanar group (transperineal targeted biopsy with transperineal systematic biopsy). PCa detection rates, lesion locations, csPCa, clinically insignificant PCa (ciPCa), and complication incidences were compared. Univariable and multivariable logistic regression models evaluated factors influencing biopsy outcomes.

**Results:**

No significant differences were observed in overall PCa detection, ciPCa, posterior lesions, or postoperative complications between biplanar and monoplanar groups. The biplanar group demonstrated a higher detection rate for anterior csPCa (P=0.025). The overall International Society of Urological Pathology grade group (ISUP GG) distributions for Prostate Imaging Reporting and Data System (PI-RADS) scores 3 to 5 were not significantly different. Logistic regression identified age and PSA levels as independent predictors of higher detection rates, while univariable analysis showed that prostate volume had a significantly smaller effect on PCa detection rates in the biplanar group compared to the monoplanar group. Postoperative complications showed no statistically significant differences.

**Conclusions:**

In conclusion, biplanar stereotactic biopsy was superior to monoplanar biopsy in detecting anterior csPCa. Both methods demonstrated no significant differences in overall PCa detection rates and safety.

## INTRODUCTION

Prostate cancer (PCa) is the second most frequently diagnosed cancer in males worldwide, ranking first in Europe and the United States (1). In recent years, the incidence of PCa has been increasing in China (2). Transrectal prostate biopsy (TRBx) primarily detects PCa in the posterior region of the prostate, but it has limited effectiveness in identifying cancers located in the anterior portion (3). Transrectal ultrasound (TRUS)-guided biopsies have been the routinely performed technique for detecting PCa, however, this method suffers from inadequate visualization of the target, leading to the underdiagnosis of clinically significant PCa (csPCa) (4).

Transperineal prostate biopsy (TPBx), by improving the sampling of the anterior prostate, has been shown to increase the detection of csPCa in patients under active surveillance, which underscores the importance of early intervention in reducing the likelihood of disease progression and associated morbidity (5). Furthermore, the development of multiparametric magnetic resonance imaging (mpMRI) and the introduction of Prostate Imaging Reporting and Data System (PI-RADS) have significantly influenced the diagnostic approach to PCa, particularly for csPCa (6). Studies indicate that MRI-TBx achieves higher detection rates of csPCa while reducing the identification of clinically insignificant prostate cancer (ciPCa) compared to systematic biopsy (7). The biplanar stereotactic biopsy method, which combines transrectal targeted biopsy with transperineal systematic biopsy, is designed to capitalize on the sensitivity of mpMRI. It was observed that prostate evasive anterior tumors were detected late and often presented with high grades (8). Both biopsy methods were performed by the same group of urologists, all with the same qualifications and expertise.

In our study, we aimed to investigate whether biplanar stereotactic biopsy could offer an advantage in detecting anterior csPCa compared to monoplanar biopsy, which combines transperineal targeted biopsy with transperineal systematic biopsy. To minimize confounding factors, we applied propensity score matching (PSM) to control for selection bias. This study has the potential to propose a prostate biopsy method that enhances the detection rate of csPCa, particularly in the anterior region.

## MATERIALS AND METHODS

### Study population

**Figure 1:**
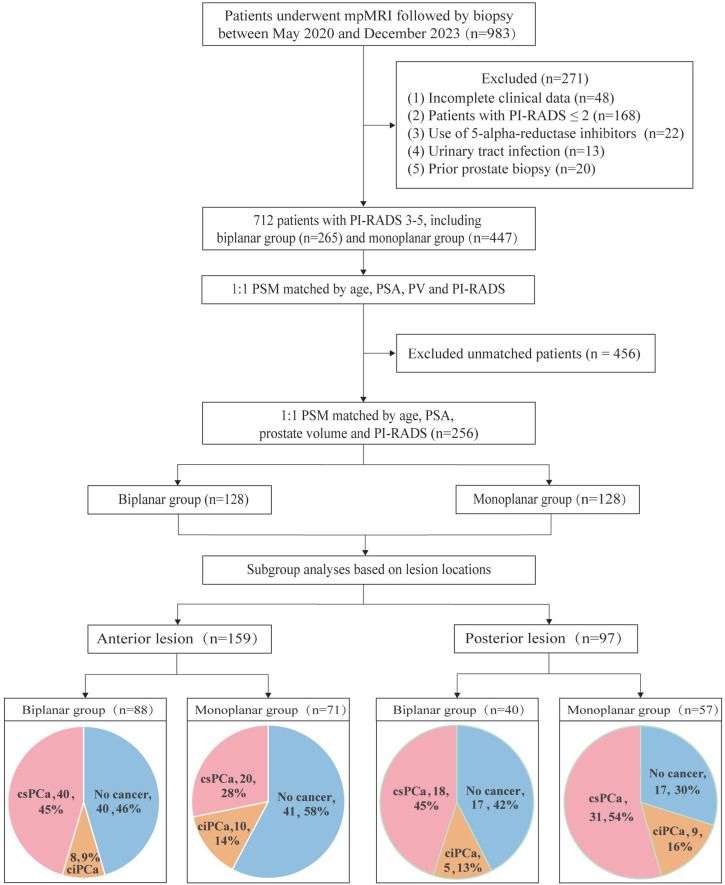
Flow chart and diagnostic accuracy for detection of anterior and posterior PCa between biplanar and monoplanar groups. PI-RADS, Prostate Imaging Reporting and Data System; ciPCa, clinically insignificant prostate cancer; csPCa, clinically significant prostate cancer.

The study retrospectively included the clinical data of 983 patients admitted to Shanghai General Hospital for prostate biopsy from May 2020 to December 2023. After applying the exclusion criteria, 271 patients were excluded, leaving a total of 712 patients. The cohort was subdivided into two groups based on the technique used at the two campus divisions of Shanghai General Hospital: 265 patients at the northern campus underwent biplanar biopsy, while 447 patients at the southern campus underwent monoplanar biopsy. Following 1:1 PSM, a final cohort of 256 patients was selected, including 128 patients in the biplanar group and 128 in the monoplanar group. Eligible patients for the study were those with the following criteria: (1) elevated prostate-specific antigen (PSA>4 ng mL-1); (2) abnormal digital rectal examination; (3) monitoring of PCa; (4) PI-RADS score greater than 2. Exclusion criteria included the following: (1) a negative multiparametric MRI (PI-RADS ≤ 2); (2) incomplete clinical data; (3) use of 5-alpha-reductase inhibitors in the past 6 months; (4) presence of a urinary tract infection or prostatitis within the preceding three months; (5) patients with prior prostate biopsy. Figure-1 illustrates the flowchart of subject selection. This study was approved by the Shanghai General Hospital Clinical Research Ethics Committee (Institutional Review Board number: IRB: K-2024-011) and registered in the Chinese Clinical Trial Register (ChiCTR2400087842).

### Clinical characteristics

In this retrospective study, all available cases were collected for comprehensive evaluation of both methods using medical records and medical coding information. The study gathered data on patient age, pre-biopsy PSA levels, MRI reports prior to biopsy, biopsy indications, and results of histopathological examination. Prostate volume was measured using TRUS and calculated with the ellipsoid volume formula: Prostate volume (mL) = (π/6) × (anterior-posterior diameter [cm]) × transverse diameter (cm) × superior-inferior diameter (cm).

### Biopsy protocol

**Figure 2:**
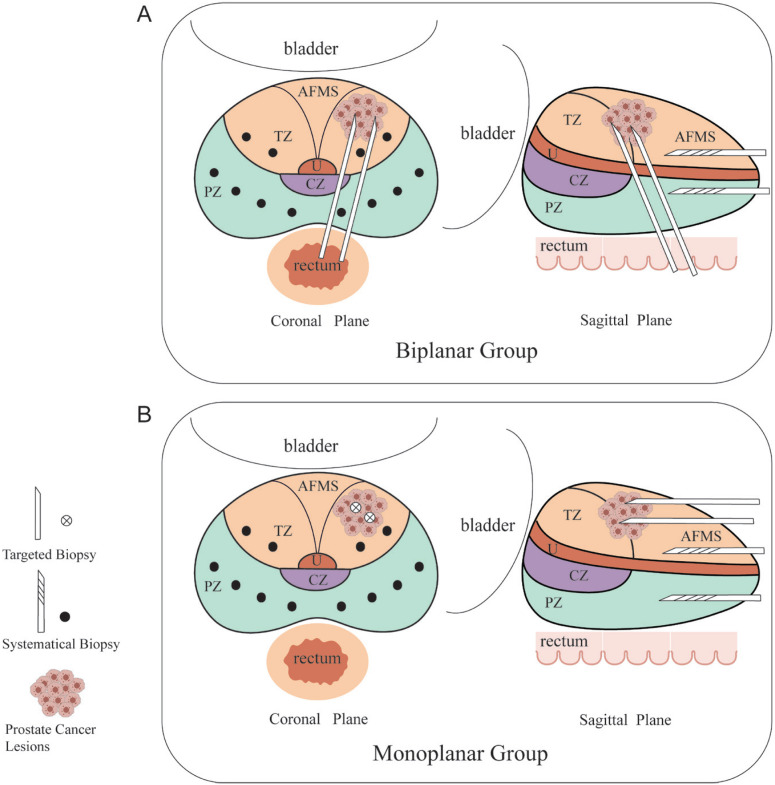
Schemes of biplanar biopsy and monoplanar biopsy. (A) Schemes of biplanar biopsy on prostatic coronal and sagittal plane. (B) Schemes of monoplanar biopsy on prostatic coronal and sagittal plane. TZ, transition zone; PZ, peripheral zone; CZ, central zone; AFMS, anterior fibrous muscle matrix; U, urethra.

**Supplementary Figure 1:**
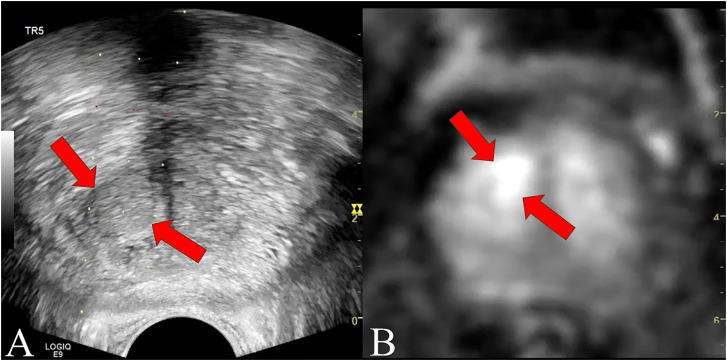
Software-assisted MRI-TRUS image fusion targeted biopsy. (a) Under transrectal ultrasound, a hypoechoic lesion is observed on the right prostate (arrows). (b) Axial diffusion weighted images (DWI) showed a large hyperintense signal lesion developed in the peripheral zone.

All patients received either biplanar stereotactic biopsy or monoplanar biopsy within one week following their mpMRI examination. A rectal needle guider was used to target suspicious cancer regions identified on mpMRI, guided by an ultrasound fusion device (GE Logic E9, GE Healthcare, Milwaukee, WI, USA). Biopsies were performed using a Bard biopsy gun equipped with disposable 18-G needles (MC1616 and MC1820, Bard Company, USA). Figure-2 illustrates the biplanar and monoplanar biopsy schemes. In the biplanar group, TRBx were performed to obtain 2-4 cores from each lesion, with assistance from MRI-TRUS image fusion software (Supplementary Figure-1). For the monoplanar group, 2-4 targeted TPBx cores were acquired from each lesion, utilizing the ultrasound device for transperineal targeted biopsy (HI VISION Preirus, Hitachi Medical Systems, Japan). After the targeted biopsy, a 12-core systematic transperineal biopsy was performed, and the standardized biopsy specimens were sent for pathological analysis. Both biopsy methods were performed by the same group of urologists, all with the same qualifications and expertise.

### Pathology and PCa diagnosis

**Supplementary Figure 2:**
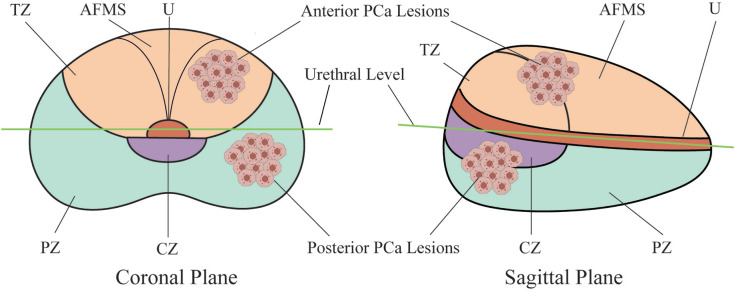
Anterior and Posterior Classification of Prostate Cancer Lesions in Coronal and Sagittal Planes. TZ, transition zone; PZ, peripheral zone; CZ, central zone; AFMS, anterior fibrous muscle matrix; U; urethra.

The pathologic evaluation of the biopsy cores, which was conducted and cross-verified in a blinded manner to reduce potential bias, reported the number of total positive cores/total cores, Gleason score, and the International Society of Urological Pathology grade group (ISUP GG). The cancer suspicious regions identified through mpMRI offered relevant information about the location of PCa lesion. Regarding the urethral level as a reference, PCa were further classified into anterior and posterior lesions (Supplementary Figure-2). Lesions identified on MRI were characterized according to the PI-RADS criteria. Histopathology results were classified using the ISUP GG, with PCa lesions scoring ISUP GG 2–5 deemed csPCa. Lesions with a maximum ISUP GG of 1 were regarded as clinically insignificant PCa (ciPCa).

### Propensity score matching

**Figure 3:**
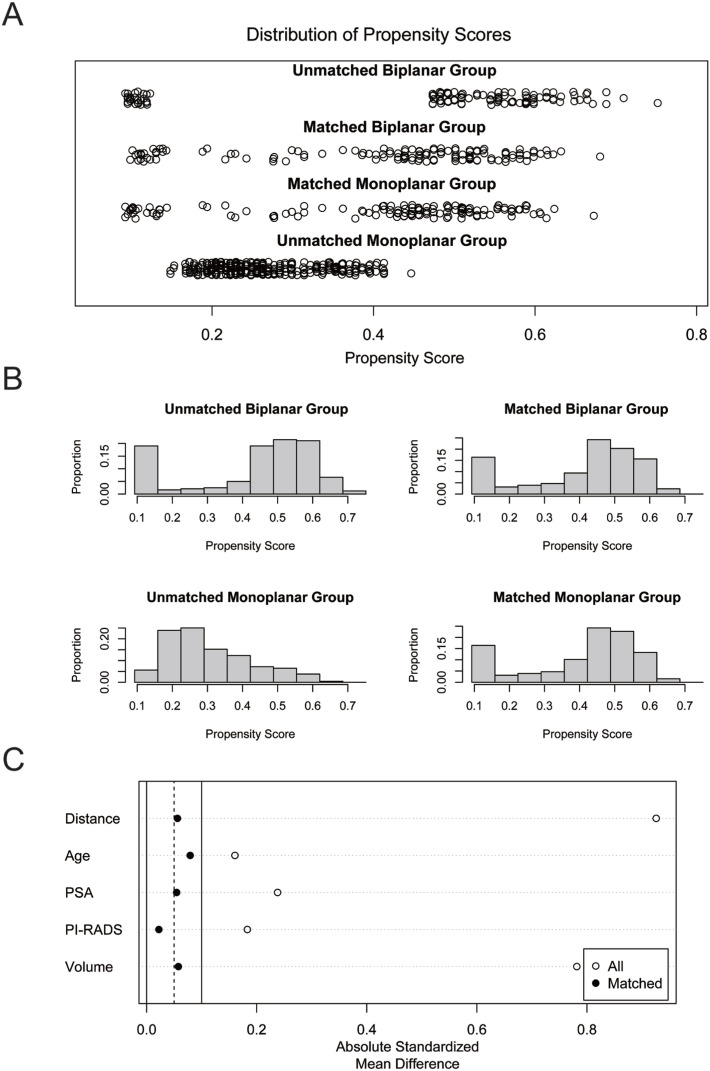
Equitable comparison of baseline covariates (age, PSA levels, prostate volume, and PI-RADS) distribution between the biplanar and monoplanar groups for diagnostic assessment. (A) Jitter plot showing individual patients' propensity score distribution for biplanar and monoplanar groups. (B) Histogram depicting the distribution of patients' propensity scores in the biplanar and monoplanar groups. (C) Baseline covariate differences before and after matching.

To minimize confounding factors and reduce bias between the two groups, the cohorts were matched using propensity scores derived from logistic regression based on patients’ age, PSA, prostate volume, and PI-RADS scores. Biplanar group patients were matched to monoplanar group patients at a 1:1 ratio using a nearest neighbor matching algorithm. A caliper width of 0.25 standard deviations of the logit of the propensity score was applied. After matching, 128 patients were selected in each group, with unmatched patients excluded from further analysis. This achieved balance across covariates as confirmed by standardized mean differences below 0.1 for all variables. Post-matching, the balance was assessed and confirmed through visual inspection of propensity score distributions and by calculating standardized mean differences. Additionally, a jitter plot of individual cases, a histogram of individual differences, and a histogram of standardized differences were generated (Figure-3). PSM was conducted using the ‘matching’ package in R version 4.2.0 (R Project for Statistical Computing) (9).

### Statistical analysis

Measurement data were expressed as mean ± standard deviation for normally distributed data, or median with interquartile range (IQR) for non-normally distributed data. Count data were expressed as frequency (n) and percentage (%) with the chi-square test, and differences were considered statistically significant at P < 0.05. To compare the clinical characteristics of patients between the groups, we employed Student's t-tests and Mann–Whitney U tests. The chi-square test, univariate and multivariate logistic regression analyses were used to compare the two groups. In both multivariable and univariable analyses, the effects were quantified using odds ratios with 95% confidence intervals. All analyses were conducted using SPSS (version 27.0) for general statistical tests, and the R package was utilized for PSM to ensure precise bias control and group balance.

## RESULTS

### Characteristics of patients

From May 2020 to December 2024, we identified 983 patients underwent mpMRI followed by prostate biopsy and 271 patients were excluded after the exclusion criteria were applied. A total of 256 patients were included in this study after PSM, with 128 men in the biplanar group and 128 men in the monoplanar group (Figure-1). The characteristics of patients in the two groups are shown in Table-1. A comparison of pre-procedure demographics, including age, PSA, and prostate volume, showed good match between the two groups (P ≥ 0.05). The differences in the number of biopsy cores taken, number of targeted biopsy cores taken, positive cores, and PI-RADS scores between the two groups were not statistically significant (P ≥ 0.05). The monoplanar group had a significantly higher mean number of positive targeted biopsy cores compared to the biplanar group (p = 0.004), indicating a statistically significant difference.

**Table 1:** Characteristics of patients according to prostate biopsy method.

		Biplanar group (n=128)	Monoplanar group (n=128)	p value
Age, year, median (IQR)		67 (62-73)	67 (62-72)	0.434 a
PSA, ng/mL, median (IQR)		9.8 (8.0-13.9)	9.2 (7.5-13.6)	0.193 b
Prostate volume, mL, mean (IQR)		52.1 (40.6-61.4)	51.0 (41.8-59.9)	0.506 b
**PI-RADS**				
	PI-RADS=3	73 (56.0%)	71 (55.5%)	0.801 c
	PI-RADS=4	18 (14.4%)	21 (16.4%)	0.602 c
	PI-RADS=5	37 (29.6%)	36 (28.1%)	0.953 c
Number of cores taken, mean (IQR)		14.37 (14-15)	14.62 (12-15)	0.162 b
Number of targeted biopsy cores taken, mean (IQR)		2.6 (2-3)	2.6 (2-3)	0.962 b
Positive cores, mean (IQR)		2.2 (1-4)	2.3 (1-3)	0.653 b
Positive targeted biopsy cores, mean (IQR)		0.2 (0-1)	0.4 (0-1)	0.054 b
Number of positive cores, n (%)		Biplanar group (n=71)	Monoplanar group (n=70)	
1		15 (21.1%)	14 (20.0%)	0.835 c
2-3		22 (31.0%)	25 (35.7%)	0.591 c
4-12		33 (46.5%)	30 (42.9%)	0.610 c
>12		1 (1.4%)	1 (1.4%)	1.000 d

SD = standard deviation; PSA = prostate-specific antigen; IQR = Interquartile Range

a student's t test;

b Mann–Whitney U test;

c chi-square test;

d Fisher's exact test

### Prostate cancer detection rate based on lesion location

**Supplementary Figure 3:**
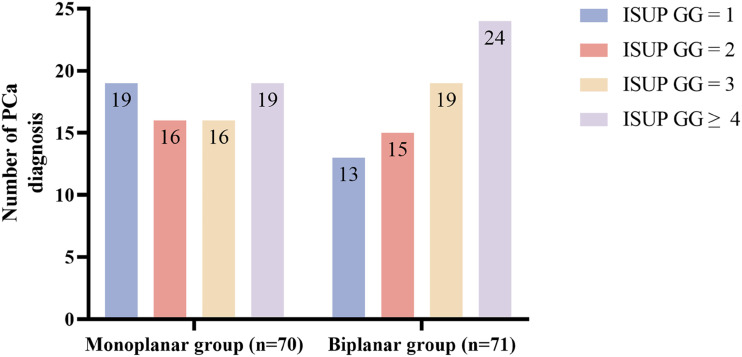
Distribution of ISUP grade group in monoplanar and biplanar groups for prostate cancer diagnosis. ISUP: International Society of Urological Pathology.

Table-2 outlines the diagnostic outcomes for both anterior and posterior prostate lesions in the biplanar and monoplanar groups. Overall, 71 (55.5%) of the patients had PCa detected in the biplanar group, of which 58 (45.3%) were csPCa. The monoplanar group identified 70 cases (54.7%) of PCa and 51 cases (39.8%) of csPCa. Comparing the biplanar group and the monoplanar group, we found no statistically significant difference in terms of the overall detection rate, csPCa detection rate and ciPCa detection rate (P > 0.05). The detection efficiency of biplanar biopsy compared to monoplanar biopsy, stratified by ISUP GG, is detailed in Supplementary Figure-3. When comparing the detection rates of anterior and posterior PCa lesions for the two biopsy methods separately, it was found that the biplanar biopsy had an advantage in detecting anterior PCa lesions. The histopathological findings of the posterior PCa lesion biopsy indicate that there is no statistically significant difference between the two biopsy groups in detecting posterior PCa lesion and ciPCa (P > 0.05). The anterior csPCa lesion detection rate in the biplanar group was 45.5%, which was higher than that in the monoplanar group (28.2%), and the difference was statistically significant (P = 0.025; Table-2).

**Table 2:** PCa detection rates stratified by PCa lesion's location.

Biopsy outcomes per subanalysis			p value a
	**Biplanar group (n=128)**	**Monoplanar group (n=128)**	
Overall detection rate	71 (55.5%)	70 (54.7%)	0.900
ciPCa	13 (10.2%)	19 (14.8%)	0.257
csPCa	58 (45.3%)	51 (39.8%)	0.376
	**Biplanar group (n=40)**	**Monoplanar group (n=57)**	
Positive biopsy rate of posterior lesion	23 (57.5%)	40 (70.2%)	0.198
Posterior ciPCa	5 (12.5%)	9 (15.8%)	0.650
Posterior csPCa	18 (45.0%)	31 (54.3%)	0.514
	**Biplanar group (n=88)**	**Monoplanar group (n=71)**	
Positive biopsy rate of anterior lesion	48 (54.5%)	30 (42.3%)	0.123
Anterior ciPCa	8 (9.1%)	10 (14.1%)	0.323
Anterior csPCa	40 (45.5%)	20 (28.2%)	0.025

PCa = prostate cancer; ciPCa = clinically insignificant prostate cancer; csPCa = clinically significant prostate cancer

a chi-square test.

To investigate whether there was a selection bias in PI-RADS scores between the two groups that could affect the detection rates of PCa, we compared the detection rates of patients in both groups under different PI-RADS scores. Supplementary Table-1 presents the breakdown of PI-RADS scores for cases identified as csPCa and ciPCa in the biplanar and monoplanar biopsy groups. There was no statistically significant difference in the distribution of patients with PI-RADS scores of 3-5 between the two groups (P > 0.05). Supplementary Table-2 presents the biopsy pathology results of patients in both groups under different PI-RADS scores, stratified by tumor location. Therefore, the advantage of biplanar biopsy in detecting anterior csPCa is not attributable to differences in PI-RADS scores between the two groups.

### ISUP distribution by PI-RADS scores

Supplementary Table-3 shows the distribution of ISUP GG in patients with different PI-RADS scores for both biplanar and monoplanar biopsy groups. The data highlight that for patients with a PI-RADS score of 5, the probability of having an ISUP GG ≥ 4 was 51.4% in the biplanar group compared to 38.9% in the monoplanar group. Despite this observed difference, no statistically significant differences were found between the two groups in terms of the distribution of ISUP GG for PI-RADS scores 3 to 5 (P > 0.05). This suggests that while the biplanar method shows a higher detection rate of more aggressive cancers (ISUP GG ≥ 4) in patients with a PI-RADS score of 5, both biopsy methods provide comparable pathological results overall for PCa detection.

### Predictors of prostate cancer detection

Multivariable and univariable logistic regression analyses identified age and PSA levels as independent predictors of higher detection rates in both the biplanar and monoplanar groups (Table-3). Patients with lower prostate volume who underwent monoplanar biopsy initially showed a higher detection rate (OR: 0.983, 95% CI: 0.902–1.071, P = 0.027). However, this association lost significance after multivariate adjustment (OR: 0.958, 95% CI: 0.862–1.066, P = 0.432). Stratifying the cohort by maximal PI-RADS score showed that detection rates of PCa were significantly higher for patients with PI-RADS scores of 4 or 5 compared to those with a score of 3 in both groups.

**Table 3:** Univariable and multivariable binary logistic regression for analyzing the effects of biopsy methods and patients’ clinical characteristics on prostate cancer detection rate

		Univariable analysis		Multivariable analysis	
		Biplanar group, OR (95% CI), p value a	Monoplanar group, OR (95% CI), p value a	Biplanar group, OR (95% CI), p value a	Monoplanar group, OR (95% CI), p value a
Age		1.026 (0.989-1.065), 0.011	1.068 (1.015-1.123), 0.012	1.071 (1.018-1.127), 0.008	1.058 (0.996-1.124), 0.047
PSA		1.032 (1.023-1.041), < 0.001	1.041 (1.026-1.055), < 0.001	1.042 (1.037-1.078), < 0.001	1.039 (1.022-1.055), < 0.001
Prostate volume		0.953 (0.874-1.039), 0.271	0.983 (0.902-1.071), 0.039	0.907 (0.805-1.021), 0.107	0.958 (0.862-1.066), 0.432
PI-RADS					
	PI-RADS=3*	-	-	-	-
	PI-RADS=4	2.677 (0.927-7.728),0.069	4.896 (1.685-14.228), 0.004	3.041 (3.010-3.163), <0.001	5.621 (1.753-18.022), 0.004
	PI-RADS=5	14.056 (4.489-44.006), <0.001	12.142 (4.186-35.217), <0.001	13.106 (13.021-13.527), 0.006	15.169 (4.689-49.074), <0.001

PSA = prostate-specific antigen; CI = confidence interval; OR = odds ratio.

a multivariable binary logistic regression;

* reference group.

### Comparison of biopsy complications

The comparison of biopsy complications between the two groups revealed no statistically significant differences in postoperative hematuria, acute urinary retention, infection, and rectal bleeding (P > 0.05). Specifically, 28 patients (21.9%) in the biplanar group and 41 patients (32.0%) in the monoplanar group presented with hematuria, with the difference in incidence not being statistically significant (P=0.067). Additionally, two patients (1.6%) in the biplanar group and three patients (2.3%) in the monoplanar group experienced acute urinary retention, with no statistically significant difference in the incidence between the two groups (P > 0.05). Importantly, no cases of infection or rectal bleeding were observed in either group.

## DISCUSSION

Since Hodge introduced the 6-core TRUS-guided biopsy as the standard for prostate biopsy, it still faced a high rate of missed diagnoses (10). To refine biopsy techniques, we aim to explore whether biplanar stereotactic biopsy can enhance PCa detection rates while minimizing complications. In this study, mpMRI-TRUS targeted biopsy was employed in both groups combined with systematic biopsy, as it offers significant advantages in detecting csPCa compared to systematic biopsy (42% vs. 26%, respectively) (11). Recent studies have shown that combining targeted biopsy with systematic biopsy significantly increased the overall detection rate of PCa (12). Additionally, MRI-TRUS targeted biopsy reduces the overdiagnosis of ciPCa, leading to less overtreatment (13). Siddiqui observed a 30% increase in csPCa detection with MRI-TRUS fusion targeted biopsy compared to systematic biopsy, while the detection rate of ciPCa decreased by 17% (14). This study utilized a 12-core transperineal systematic biopsy, which has been shown to improve the detection rate of PCa without increasing complications compared to the 6-core transperineal systematic biopsy (15).

The results of our study showed no significant difference in the overall detection rate of PCa, ciPCa, and posterior PCa between the biplanar and monoplanar groups (P > 0.05). Specifically, systematic biopsies in both groups were performed via the transperineal route. Prostate evasive anterior tumor syndrome describes anteriorly located tumors that can evade standard biopsy techniques but are detectable through MRI, highlighting the need for further examination to rule out PCa (16). Given these challenges in detecting anterior lesions, TPBx offers greater flexibility and accuracy, allowing for more extensive sampling of the peripheral zone tissue and a higher detection rate of csPCa in the anterior prostate, which might be missed by TRBx. Pepe et al. demonstrated that the transperineal route achieved a markedly higher detection rate of anterior zone csPCa compared to the transrectal approach, with rates of 93.3% versus 25% (17). Therefore, the use of TPBx for systematic biopsy in both groups in this study helps to mitigate the potential bias of missing anterior lesions with TRBx, strengthening the credibility of the biplanar biopsy's advantage in detecting anterior lesions.

In comparing the detection rates of anterior csPCa between the two groups of PSM-matched patients, the biplanar approach demonstrated superiority over the monoplanar biopsy method (P < 0.05). This difference may be attributed to the advantages of biplanar stereotactic biopsy, which combines transperineal and transrectal approaches, allowing biopsies in both transverse and sagittal planes, thus providing a broader sampling area. Both systematic and targeted biopsies encounter difficulties in detecting apical lesions, but a combined biopsy approach can significantly enhance detection rates of PCa (18). Targeted biopsy frequently misses cancers in the posterior region, while TRUS-guided biopsy often fails to identify lesions located in the anterior region (19). The biplanar biopsy method combines transrectal and transperineal approaches, offering broader access to different prostate regions and improving detection, particularly for hard-to-reach anterior lesions. The transrectal approach may be more effective in patients with prostate volumes of 30–80 mL and advanced stages (T3–T4), whereas the transperineal approach shows greater efficacy in detecting cancers at earlier stages (T1–T2) (20). The increased sampling area of the biplanar method, similar to the regional saturation biopsy approach shown to improve the detection of clinically significant prostate cancer by enhancing coverage of suspected regions, potentially reduces the chance of missing significant cancerous lesions, leading to more accurate diagnoses (21). Additionally, the biplanar biopsy technique incorporates transrectal image fusion-guided biopsy, enabling the clinician to integrate TRUS and mpMRI images for precise lesion localization in the coronal plane, thereby enhancing the accuracy of targeting. In contrast, the monoplanar approach, limited to coronal plane biopsies, lacks sagittal plane sampling, which complicates precise localization of anterior lesions using transrectal ultrasound alone (22). This limitation requires exceptional biopsy skills and accurate spatial judgment by urologists, increasing the risk of localization errors and leading to a lower detection rate of clinically significant PCa (23). This makes the biplanar biopsy an effective screening method for anterior PCa when indicated by mpMRI.

The results of both univariable and multivariable logistic regression analyses identified age and PSA levels as significant independent predictors of PCa detection in both biplanar and monoplanar groups. Interestingly, in the monoplanar group, lower prostate volume initially appeared to be associated with higher detection rates, but this lost significance after multivariate adjustment, suggesting other factors like age and PSA were more impactful (24, 25). Furthermore, when stratifying by PI-RADS scores, patients with PI-RADS 4 or 5 had notably higher detection rates compared to those with a score of 3 in both biopsy groups (26). The strong predictive value of PI-RADS scores, aligns with findings from prior studies, including the recent validation of the BCN-MRI PM, which demonstrated that mpMRI could reliably predict csPCa, further emphasizing their utility in PCa detection (27, 28). While both biopsy methods showed similar predictive trends, the data suggest that a more individualized approach considering patient-specific factors such as age, PSA levels, and PI-RADS scores could optimize diagnostic accuracy.

There was no significant difference in the incidence of hematuria or urinary retention between the two groups following biopsy (P > 0.05). This aligns with the findings of the recent ProBE-PC trial, which demonstrated that there were no significant differences in postoperative complication rates between TRBx and TPBx (29). In the biplanar group, transrectal targeted biopsy with only 2-4 cores per lesion was employed, reducing the likelihood of intestinal flora entering the bloodstream via the intestinal wall and prostate tissue, suggesting a safer procedure with a lower risk of infectious prostatitis, as seen in the lower complication rates of TPBx compared to TRBx (30). This substantial reduction in infectious complications significantly improved the safety profile of the biopsy. The remarkably low postoperative complication rates observed in this study suggest that both methods were comparably safe and effective for urological procedures.

This investigation has several limitations. Firstly, as a retrospective study, it may be subject to inherent limitations. Although we attempted to minimize confounding factors such as age, PSA, and prostate volume through PSM, the process could not fully eliminate selection bias. A prospective study and blinded data processing would be valuable in further validating our result. Secondly, this was a single-center study with limited PSM samples. Future studies with larger sample sizes and multicenter designs are needed to validate these findings. Finally, there may be pathological verification bias, particularly in patients diagnosed as cancer-free, since biopsy outcomes were used as the reference rather than surgical pathology results.

## CONCLUSION

Biplanar stereotactic biopsy demonstrates a notable advantage over monoplanar biopsy in the detection of anterior csPCa lesions. Both biplanar stereotactic biopsy and transperineal monoplanar biopsy effectively detect PCa and ciPCa, while maintaining comparable safety. In both biopsy groups, age and PSA levels were key independent predictors of PCa detection, with the biplanar biopsy showing less impact from prostate volume compared to the monoplanar biopsy. Biplanar stereotactic biopsy may serve as an effective screening approach for detecting anterior csPCa identified by mpMRI.

## Data Availability

Data for this study can be accessed by contacting the corresponding author with a reasonable request.

## ABBREVIATIONS

ISUP: = International Society of Urological Pathology
PI-RADS: = Prostate Imaging Reporting and Data System
PCa: = Prostate cancer; csPCa: Clinically significant prostate cancer
ciPCa: = Clinically insignificant prostate cancer
TRUS: = Transrectal Ultrasound-Guided
TPB: = Transperineal biopsy
TRB: = Transrectal biopsy
mpMRI: = Multiparametric Magnetic Resonance Imaging
PSA: = Prostate-specific antigen
IQR: = Interquartile Range
OR: = Odds ratio
CI: = Confidence interval
SD: = Standard deviation

## APPENDIX

**Supplementary Table 1:** Prostate cancer detection rates of three PI-RADS scores using different biopsy methods.

PI-RADS	Core Positive	Biplanar group (n=128)		Monoplanar group (n=128)		p value
3	No PCa	58.9%	(46/73)	66.2%	(47/71)	0.690^a^
	PCa	37.0%	(27/73)	33.8%	(24/71)	
	ciPCa	16.4%	(12/73)	18.3%	(13/71)	0.767^a^
	csPCa	20.5%	(15/73)	15.5%	(11/71)	0.430^a^
4	No PCa	38.9%	(7/18)	28.6%	(6/21)	0.520^b^
	PCa	61.1%	(11/18)	71.4%	(15/21)	
	ciPCa	16.7%	(3/18)	19.0%	(4/21)	0.110^b^
	csPCa	38.9%	(7/18)	52.4%	(11/21)	0.523^b^
5	No PCa	10.8%	(4/37)	13.9%	(5/36)	0.736^b^
	PCa	89.2%	(33/37)	86.1%	(31/36)	
	ciPCa	2.7%	(1/37)	5.6%	(2/36)	0.615^b^
	csPCa	86.5%	(32/37)	80.6%	(29/36)	0.494^a^

PI-RADS = Prostate Imaging Reporting and Data System; PCa = prostate cancer; ciPCa = clinically insignificant prostate cancer; csPCa = clinically significant prostate cancer

a chi-square test

b Fisher's exact test

**Supplementary Table 2:** Prostate cancer detection rates of three PI-RADS scores in anterior and posterior lesions.

PI-RADS	Core Positive	Anterior Lesions (n=159)		p value	posteriors Lesions (n=97)		p value
		Biplanar group	Mono-planar group		Biplanar group	Mono-planar group	
3	PCa	35.4% (17/48)	26.1% (12/46)	0.328^a^	40.0% (10/25)	48.0% (12/25)	0.569^a^
	csPCa	20.8% (10/48)	10.9% (5/46)	0.187^a^	20.0% (5/25)	21.0% (6/25)	0.733^a^
4	PCa	54.5% (6/11)	50.0% (3/6)	1.000^b^	71.4% (5/7)	80.0% (12/15)	1.000^b^
	csPCa	54.5% (6/11)	33.3% (2/6)	0.620^b^	71.4% (5/7)	60.0% (9/15)	1.000^b^
5	PCa	86.2% (25/29)	78.9% (15/19)	0.695^b^	100% (8/8)	94.1% (16/17)	1.000^b^
	csPCa	82.8% (24/29)	68.4% (13/19)	0.304^b^	100% (8/8)	94.1% (16/17)	1.000^a^

PI-RADS = Prostate Imaging Reporting and Data System; PCa = prostate cancer; ciPCa = clinically insignificant prostate cancer; csPCa = clinically significant prostate cancer

a chi-square test

b Fisher's exact test

**Supplementary Table 3:** ISUP of mono-planar and biplanar group according to the PI-RADS of the patients.

PI-RADS	Biplanar group						Mono-planar group						Total
	No PCa	ISUP=1	ISUP=2	ISUP=3	ISUP≥4	Patients	No PCa	ISUP=1	ISUP=2	ISUP=3	ISUP≥4	Patients	
3	46 (63.0%)	12(16.4%)	8(11.0%)	4(5.5%)	3(4.1%)	73	47(66.2%)	13(18.3%)	4(5.6%)	3(4.0%)	4(5.6%)	71	144
4	7(38.9%)	0(0%)	4(22.2%)	5(27.8%)	2(11.1%)	18	6(28.6%)	4(19.0%)	6(28.6%)	4(19.0%)	1(4.8%)	21	39
5	4(10.8%)	1(2.7%)	3(8.1%)	10(27.0%)	19(51.4%)	37	5(13.9%)	2(5.6%)	6(16.7%)	9(25.0%)	14(38.9%)	36	73
Total	54(43.2%)	13(10.4%)	15(8.1%)	19(15.2%)	24(19.2%)	128	61(46.6%)	19(14.5%)	16(12.2%)	16(12.2%)	19(14.5%)	128	256

ISUP = International Society of Urological Pathology; PI-RADS = Prostate Imaging Reporting and Data System
